# Impact of Maternal Intrapartum Antibiotics, and Caesarean Section with and without Labour on *Bifidobacterium* and Other Infant Gut Microbiota

**DOI:** 10.3390/microorganisms9091847

**Published:** 2021-08-31

**Authors:** Yuan Yao Chen, Xin Zhao, Wolfgang Moeder, Hein M. Tun, Elinor Simons, Piushkumar J. Mandhane, Theo J. Moraes, Stuart E. Turvey, Padmaja Subbarao, James A. Scott, Anita L. Kozyrskyj

**Affiliations:** 1 Department of Pediatrics, Faculty of Medicine and Dentistry, University of Alberta, Edmonton, AB T6G 1C9, Canada; yuanyao@ualberta.ca (Y.Y.C.); xzhao1@ualberta.ca (X.Z.); heinmtun@hku.hk (H.M.T.); mandhane@ualberta.ca (P.J.M.); 2 Dalla Lana School of Public Health, University of Toronto, Toronto, ON M5T 1R4, Canada; wolfgang.moeder@utoronto.ca (W.M.); james.scott@utoronto.ca (J.A.S.); 3 HKU-Pasteur Research Pole, School of Public Health, University of Hong Kong, Hong Kong SAR 999077, China; 4 Department of Pediatrics and Child Health, Children’s Hospital Research Institute of Manitoba, University of Manitoba, Winnipeg, MB R3E 0J9, Canada; Elinor.Simons@umanitoba.ca; 5 Department of Pediatrics and Physiology, Hospital for Sick Children, University of Toronto, Toronto, ON M5G 1X8, Canada; theo.moraes@sickkids.ca (T.J.M.); padmaja.subbarao@sickkids.ca (P.S.); 6 Department of Pediatrics, Child and Family Research Institute, BC Children’s Hospital, University of British Columbia, Vancouver, BC V5Z 4H4, Canada; sturvey@bcchr.ca

**Keywords:** *Bifidobacterium*, *Bifidobacterium longum* subsp. *infantis*, infant gut microbiota, caesarean section, maternal intrapartum antibiotics, labour, breastfeeding

## Abstract

Background and Aims: Few studies consider the joint effect of multiple factors related to birth, delivery mode, intrapartum antibiotic prophylaxis and the onset of labour, on the abundance of *Bifidobacterium* and the quantity of this genus and its species *Bifidobacterium longum* subsp. *infantis* in the infant gut microbiota. We implemented such a study. Methods: Among 1654 Canadian full-term infants, the gut microbiota of faecal samples collected at 3 months were profiled by 16S rRNA sequencing; the genus *Bifidobacterium* and *Bifidobacterium longum* subsp. *infantis* were quantified by qPCR. Associations between *Bifidobacterium* and other gut microbiota were examined by Spearman’s rank correlation. Results: Following vaginal birth, maternal IAP exposure was associated with reduced absolute quantities of bifidobacteria among vaginally delivered infants (6.80 vs. 7.14 log_10_ (gene-copies/g faeces), *p* < 0.05), as well as their lowered abundance relative to other gut microbiota. IAP differences in infant gut bifidobacterial quantity were independent of maternal pre-pregnancy body-mass-index (BMI), and remarkably, they were limited to breastfed infants. Pre-pregnancy BMI adjustment revealed negative associations between absolute quantities of bifidobacteria and CS with or without labour in non-breastfed infants, and CS with labour in exclusively breastfed infants. Significant correlations between *Bifidobacterium* abundance and other microbial taxa were observed. Conclusions: This study documented the impact of the birth mode and feeding status on the abundance of gut *Bifidobacterium*, and pointed to the important ecological role of the genus *Bifidobacterium* in gut microbiota due to its strong interaction with other gut microbiota in early infancy.

## 1. Introduction

How we are born shapes the development of our gut microbiota. Caesarean section (CS) birth alters the gut microbiota of the young infant with its signature influence being the substantial reduction in the abundance of Bacteroidetes species [1,2]. Bifidobacteria are also reportedly reduced in number with CS vs. vaginal delivery, as early as 3–5 days after caesarean birth [3], but these differences are less apparent 6 months later [2,4]. Mother’s milk, specifically its constituent human milk oligosaccharides (HMOs), was credited with an expansion of bifidobacteria [5]. The original source of bifidobacterial species depletion in infancy was attributed to surgical interference of maternal microbiota transfer during birth [6,7]. Interestingly, bifidobacteria are minimally depleted immediately after birth in the meconium (first stool) of infants born by caesarean, with or without labour, compared to vaginal delivery [8,9].

Whereas the imprint of caesarean section is substantive, other birth events also influence the infant gut microbial composition during the first year of life. Although not often reported, prolonged labour during birth is associated with reduced abundance of *Bifidobacterium* [10]. As vaginal microbes are considered one of the primary sources of “seeding” or transferring of microorganisms from mother to newborn [6,11], comparing the microbiota between infants delivered by CS with and without labour is worthy of study. In addition, newborns are exposed to maternal intrapartum antibiotic prophylaxis (IAP) according to clinical practice guidelines for the prevention of maternal infection associated with CS delivery or prophylaxis of vaginal Group B *Streptococcus* (GBS) in North America [12]. Within 2 days of IAP, newborn gut microbiota are depleted in bifidobacteria [13]. However, the impact of maternal IAP exposure on the abundance of *Bifidobacterium* at 3–4 months was not found in a subpopulation of the CHILD birth cohort [14]. In this study and others, whole microbial community profiling of infant faecal samples was conducted by 16S sequencing, in which universal primers under-represent the presence of bifidobacteria [15,16].

Members of the genus *Bifidobacterium* are pioneer gut colonizers and are considered foundational microbiota members that influence the early-life gut microbial community and exert positive effects on host health [17]. In addition to birth, infant diet shapes the gut microbiota of early life, with the greatest difference between breast-fed and formula-fed infants being the abundance of *Bifidobacterium* [18,19]. Breast milk is a rich source of glycans, dominated by HMOs, which selectively encourages the growth of *Bifidobacterium* [5,20]. Indeed, formula-feeding, which leads to a deficiency of *Bifidobacterium*, is associated with detrimental health outcomes, such as overweight, allergies and asthma [21,22].

Although the influence of breastfeeding in promoting *Bifidobacterium* in gut microbiota is well-known, factors related to birth, such as maternal IAP exposure and CS with labour, have been less studied. The objective of our paper was to fill this gap in the literature and build on findings from our previous research [14] to determine the association between maternal IAP, CS with and without labour, and genus *Bifidobacterium* and other gut microbial taxa in early infancy. Faeces were collected from infants at 3 months of age, and profiled by 16S rRNA sequencing, as well as quantitative PCR (qPCR), to provide an accurate measure of the absolute quantities of bifidobacteria. Breastfeeding status was taken into account.

## 2. Methods

### 2.1. Study Design

This study includes a subset of 1654 infants enrolled in the Canadian Healthy Infant Longitudinal Development (CHILD) Study between 2009 and 2012 (www.childstudy.ca, accessed on 1 May 2021), in which 3-month infant faecal samples were additionally profiled for *Bifidobacterium* by qPCR. Mothers of studied infants were recruited in their second or third trimester of pregnancy and their newborns enrolled if they were a singleton live birth at 35 weeks of gestation with a birth weight of 2500 g. In vitro fertilized births were excluded to avoid multiple gestation or preterm births. Hospital birth records provided information on sex, mode of delivery (vaginal, elective and emergency caesarean), duration of the first and second stage of labour, and maternal IAP. Women colonized with vaginal GBS or delivered by caesarean section received intrapartum administration of penicillin and cefazolin, respectively. These data were utilized to classify infants into mutually exclusive groups as follows: vaginal delivery with no IAP, vaginal delivery with IAP, CS delivery with no labour and CS delivery with labour. CS delivery was also defined clinically as elective CS (also known as scheduled or planned or without labour) and emergency CS, as per our previous study [14]. Although emergency CS delivery is commonly performed after the onset of labour [23], some emergency caesareans are performed before labour due to fetal distress. Data on maternal characteristics, including ethnicity, age, education level and prenatal smoking, as well as the infant characteristics, such as gestational age, breastfeeding status and infant antibiotic use before 3 months, were obtained from standardized questionnaires completed by mothers. Maternal pre-pregnancy body-mass-index (BMI) and prepregnancy weight status was measured as previously described [24]. All infants provided faecal samples at 3 months, which were analyzed by 16s rRNA sequencing and targeted qPCR to detect total bifidobacteria and *Bifidobacterium longum* subsp. *infantis*. A directed acyclic graph (DAG) was generated to illustrate putative relations between the birth mode and infant gut microbiota at 3 months using the daggity.net program (Accessed date: 01 May 2021) [25], which allows the selection of the minimum set of covariates required for adjustment [26,27] (Figure S1). Potential confounding factors selected by DAG (pre-pregnancy BMI, maternal ethnicity and education) were adjusted in the multivariable regression models if they caused a 15% change in the beta-coefficient for birth mode. As infant antibiotic treatment can be a confounding factor or a mediator (as shown in the DAG), we also tested this variable in models with the 15% estimate rule. Only maternal prepregnancy BMI met the 15% criterion and was adjusted in regression models. This microbiome study of the CHILD cohort was approved by the Human Research Ethics Boards of the University of Alberta (Pro00010073; 1 November 2020).

### 2.2. Faecal Microbiota Analysis

Sample collection, DNA isolation and amplification, 16S rRNA sequencing and taxonomic classification were performed as previously described [14,28]. Briefly, faecal samples of 5–10 g, collected from infant diapers during home-visits conducted at 3–4 months of age by a research assistant or parents according to an approved protocol, were refrigerated after collection, aliquoted and stored at −80 °C until analysis. Genomic DNA was isolated from frozen stool samples (80 to 200 mg) using the QIAamp DNA Stool Mini kit (Qiagen, Venlo, the Netherlands) according to the manufacturer’s instructions. The V4 hypervariable region of the bacterial 16S rRNA gene was amplified by PCR with universal bacterial primers: V4-515f: 5′-AAT GAT ACG GCG ACC ACC GAG ATC TAC ACT ATG GTA ATT GTG TGC CAG CMG CCG CGG TAA-3′, V4-806r: 5′-CAA GCA GAA GAC GGC ATA CGA GAT XXXXXXXXXXXX AGT CAG TCA GCC GGA CTA CHV GGG TWT CTA AT-3′. PCR products were paired-end sequenced on an Illumina MiSeq platform. Following a QIIME pipeline (v1.6.0, qiime.org, accessed on 1 May 2021) [29], forward and reverse reads were assembled, demultiplexed and filtered against the GreenGenes bacterial reference database (v13.8) [30] to discard the sequences with less than 60% similarity. Resultant sequences were clustered using the closed—picking algorithm at 97% similarity by USEARCH, and taxonomic assignment was carried out using the RDP classifier [31]. Finally, microbiota data were rarefied to 13,000 sequences per sample and relative abundances were calculated.

### 2.3. Quantitative PCR (qPCR) for Total Bifidobacteria and Bifidobacterium longum *subsp.* Infantis Detection

A quantitative PCR assay of DNA isolated from infant faecal samples was conducted following a published protocol [32]. To minimize differential inhibitory effects due to variable concentrations of genomic template DNA in qPCR, all template DNA samples were first normalized by dilution to 1 ng/μL. Then, each multiplex assay was prepared to contain the 1X QuantiNova Multiplex PCR Kit (Qiagen, Venlo, the Netherlands), 0.4 μM of each primer, 0.25 μM of each probe and 1 μL (1 ng/μL) of sample DNA in a final volume of 20 μL. qPCR cycling conditions were as follows: initial denaturation for 2 min at 95.0 °C, 40 cycles of denaturation for 5 s at 95 °C and annealing/extension/reading for 20 s at 60 °C. Oligonucleotides were acquired from IDT (Integrated DNA Technologies Inc, Coralville, IA, USA) and reactions were performed on the MiniOpticonTM Real-Time PCR System (Bio-Rad, Hercules, CA, USA). The primers used were specific for *Bifidobacterium longum* subsp. *infantis* (Blon0915), total bifidobacteria (Bif) and total bacteria (Total) [33,34] (Table 1).

A standard curve was created and employed to determine the efficiency of the primers and probes by performing five 1:10 serial dilutions of *B. longum* subsp. *infantis* (DSM-20088) genomic DNA starting at 1 ng/μL. We calculated the lower limit of detection for the multiplex assay to be 2.8 × 10^−6^ ng of DNA or 1 gene-copy of *B. longum* based on the amplification data from the serial dilution and the non-template control. Because each template sample represented a different starting mass of stool, the limit of quantification for the analysis was variable from sample-to-sample, and ranged from 200 to 2 × 10^9^ gene-copies/g stool (*B. longum* subsp. *infantis*) and 40 to 4.5 × 10^9^ gene-copies/g stool (total bifidobacteria). The relative abundance of *B. longum* subsp. *infantis* and total bifidobacteria was calculated as the percentage of respective taxa (gene-copies per gram stool) relative to the total bacteria (gene-copies per gram stool) quantified by qPCR. This relative abundance measure of *Bifidobacterium* was plotted against that of 16S relative abundance to determine the extent of agreement. In calculating qPCR-based abundance, almost every DNA product amplified from target 16S rDNA regions resulted in a much larger denominator and lower abundance values than 16S relative abundance. Overall (Figure S2), qPCR-based relative abundance was significantly correlated with 16S-based relative abundance, although some variations across the two methods were apparent. We found that qPCR-based relative abundance was smaller than 16S-based relative abundance in the lower range of abundance values, whereas qPCR-based relative abundance was greater than 16S-based relative abundance values in the higher range of abundance. Herein, the values of gene-copies/g stool are referred to as absolute quantities to distinguish them from calculated relative abundance values.

### 2.4. Statistical Analysis

The statistical analysis was performed using R (version 3.5.1) [35] within RStudio (version 1.2.1335) [36]. In this study, 16S microbial taxa at the phylum and genus levels, with an average relative abundance >0.05%, were subjected to downstream statistical analysis. Comparisons of categoric variables of maternal and infant characteristics were carried out using Fisher’s exact tests. To determine the impact of maternal IAP and mode of delivery on infant gut microbiota at 3 months, alpha-diversity (microbial differences within samples) was calculated with four standard indices: observed OTUs, Chao1, Shannon and PD whole tree. Beta-diversity (microbial differences between samples) was analyzed by the permutational multivariate ANOVA (PERMANOVA) and a test for multivariate homogeneity of group dispersions (PERMDISP), based on Bray-Curtis dissimilarity matrices with 1000 permutations. Both analyses were conducted using QIIME2.

To determine the modifying effect of breastfeeding, statistical analyses were conducted following stratification by breastfeeding status (exclusive, partial and non-breastfeeding). The discriminative 16S taxonomic biomarkers of infant gut microbiota were identified using linear discriminant analysis (LDA) effect size (LEfSe) [37], and significant differences were measured by an LDA score > 2 and *p* value < 0.05, using vaginal delivery with no IAP as the reference group. The associations between *Bifidobacterium* and other gut microbial genera were examined using Spearman’s rank correlation. False discovery rates (FDR) were controlled using Benjamini–Hochberg adjustment [38], with ‘FSA’ package in R [39]. FDR-corrected *p*-values < 0.1 were considered significantly different and displayed. Statistically significant differences in 16S alpha diversity indices, relative and absolute quantity of total bifidobacteria measured by qPCR and 16S rRNA sequencing were determined using the Kruskal-Wallis test; and pairwise comparisons were conducted by Dunn’s post hoc test for multiple comparison with Benjamini-Hochberg *p* value adjustments. Independence of association between birth mode and absolute quantity of *Bifidobacterium* was determined by univariable and multivariable linear regressions. To improve normality of data, the absolute quantity of genus *Bifidobacterium* was Box–Cox transformed prior to linear regression [40]. Logistic regression modelling was utilized to assess the association between the birth mode and the colonization of *B. longum* subsp. *Infantis*.

## 3. Results

In this population cohort of 1654 full-term infants at 3 months of age (mean = 3.57, SD = 1.01), 53.75% were male. The mean gestational age was 39 weeks (mean = 39.23, SD = 1.40). Over half of their mothers were of Caucasian ethnicity (74.30%), obtained a university degree (56.83%) and did not smoke (95.11%), and were between the ages of 30 and 39 years (65.48%). In addition, 55.08% and 27.27% of infants were exclusively and partially breastfed, and 17.65% of infants were formula-fed. Further, 876 infants were born vaginally without maternal IAP exposure (52.96%) and 375 were born vaginally following maternal IAP (22.67%); 10.40% (*n* = 172) and 13.97% (*n* = 231) of infants were delivered by CS with and without labour, respectively. Compared with infants delivered vaginally without IAP exposure, a greater proportion of CS delivered infants, particularly those who did not experience labour, was born to mothers who were Asian, with age over 30, and overweight and obese pre-pregnancy; more infants delivered by CS with no labour were male, and partial or non-breastfed than infants delivered vaginally without IAP exposure. Meanwhile, the lowest level of postnatal infant antibiotic use was observed among vaginally delivered infants without IAP, while infants delivered by CS with labour had the highest level of postnatal antibiotic use (Table S1).

### 3.1. Quantification of Bifidobacteria and B. longum *subsp.* infantis According to a Birth Mode

**Figure 1:**
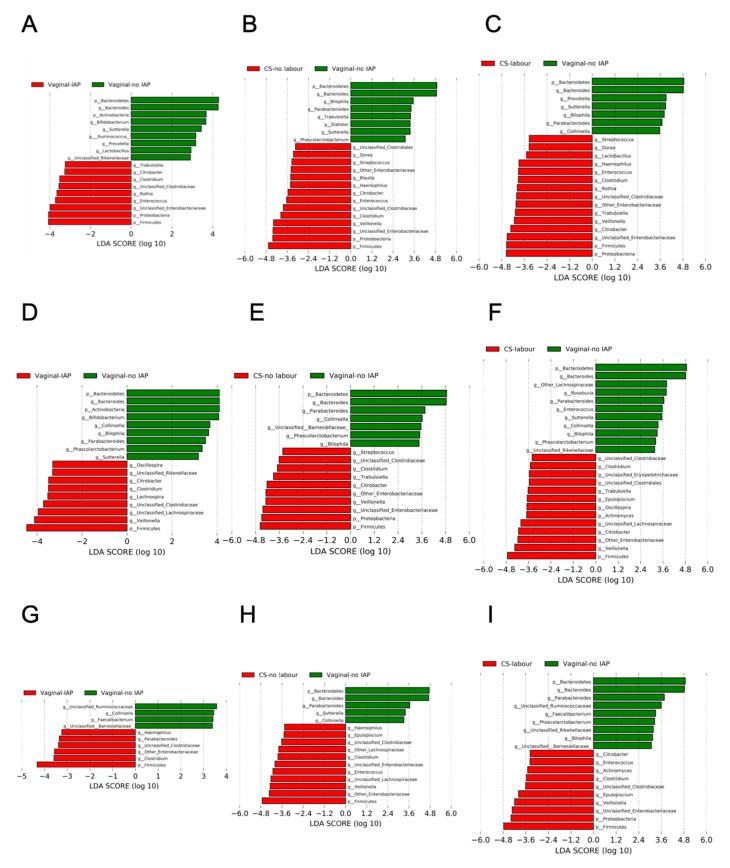
Linear discriminant analysis of 16S gut microbiota by a birth mode within exclusively breastfed (**A**–**C**), partially breastfed (**D**–**F**) and non-breastfed (**G**–**I**) infants at 3 months.

Against a backdrop of other changes to gut microbiota, 3-month old infants with maternal IAP exposure during vaginal birth had reduced relative abundance of the phylum Actinobacteria and its the actinobacterial genus *Bifidobacterium* (Figure S3). Specifically, bifidobacteria were less abundant in IAP-exposed infants than those of unexposed infants who were exclusively or partially breastfed, while this difference was not significant in non-breastfed infants (Figure 1A,D). As noted, total bifidobacteria and *B. longum* subsp. *infantis* in faecal samples were further quantified by qPCR as absolute quantities in faecal samples. Maternal IAP exposure, or CS with or without labour, were associated with lower absolute quantities of genus *Bifidobacterium* species compared to vaginal birth with no IAP (Table S2). When comparisons were conducted within infant feeding strata in our study, statistically significant reductions in bifidobacterial quantity were observed between IAP and no IAP in vaginally delivered infants who were exclusively (6.83 vs. 7.14 log_10_ (gene-copies/g faeces), *p* < 0.05) and partially breastfed (6.75 vs. 7.25 log_10_ (gene-copies/g faeces), *p* < 0.05, Table S2). Non-breastfed infants had lower absolute quantities of bifidobacteria at 3 months, especially if they were delivered by CS with or without labour (6.71 or 6.52 log_10_ (gene-copies/g faeces) vs. 6.94 log_10_ (gene-copies/g faeces) for vaginal delivery without IAP).

In accordance with results from the LEfSe analysis of 16S abundance data (Figure 1), vaginal delivery with maternal IAP exposure decreased the relative qPCR abundance of bifidobacteria. The median values of relative abundance were 1.50% and 1.13% among IAP-exposed infants, compared with 3.31% and 3.87% among unexposed infants who were exclusively and partially breastfed, respectively (Table S2). CS delivery, irrespective of labour presence or breastfeeding status, had no significant influence on the relative 16S or qPCR abundance of bifidobacteria when compared to vaginal delivery without IAP (Figure 1B,C,E,F,H,I, Table S2). When testing the clinical categories of elective and emergency CS delivery, which were employed in our previous study [14], some differences in the relative abundance of qPCR bifidobacteria were found (Table S2). With exclusive breastfeeding, elective CS delivered infants had 5.29% of *Bifidobacterium*, while infants born by emergency CS had 1.63% of this genus in their faecal gut microbiota (*p* < 0.05, measured by qPCR, Table S2). The relative abundance of qPCR bifidobacteria was also higher in elective CS vs. vaginal IAP. These differences were no longer evident when CS delivery was re-grouped as CS with and without labour (Table S2). No statistically significant differences were found when CS without labour was compared to elective CS birth, or CS with labour to emergency CS birth (Table S2).

#### Independent Associations between *Bifidobacteria*, *B. longum* subsp. *infantis* and Birth Mode

**Figure 2:**
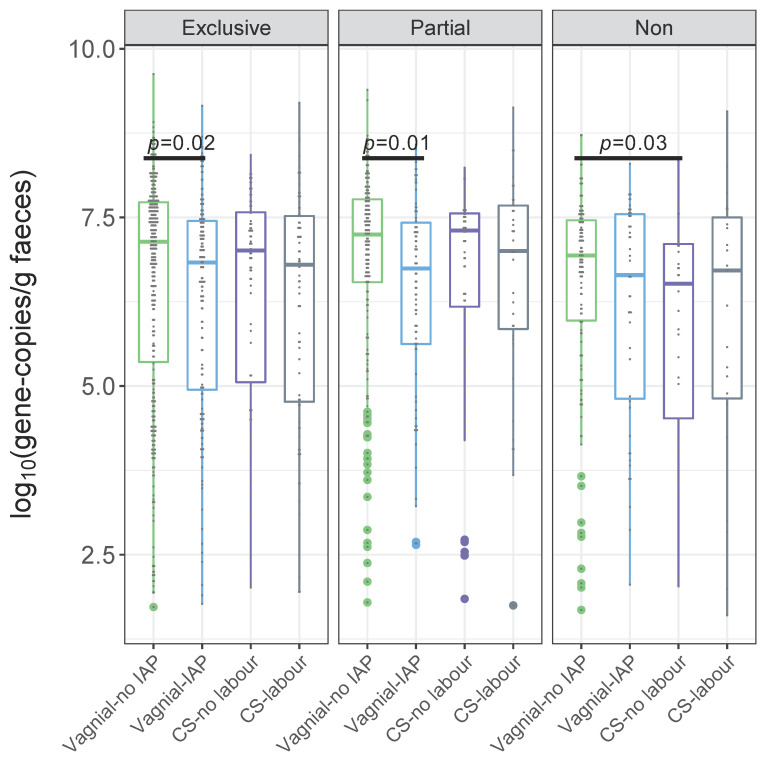
Birth mode comparisons of the absolute quantity of genus *Bifidobacterium* by qPCR within exclusively breastfed, partially breastfed and non-breastfed infants at 3 months of age. *p* values are from crude linear regression models of the Box–Cox transformed absolute quantity of genus *Bifidobacterium*. Vaginal-no IAP is the reference group.

In linear regression models predicting the Box–Cox transformed absolute quantity of bifidobacteria, crude beta-coefficients were statistically significant for vaginal delivery with maternal IAP exposure (*p* < 0.05 for all infants, Table 2), indicating a lower absolute quantity of gut bifidobacteria vs. the reference group of vaginal birth with no IAP. This association persisted following adjustment for maternal BMI (*p* < 0.05, Table 2). For comparisons conducted within infant feeding strata, statistically significant reductions in bifidobacterial quantity were observed between IAP and no IAP in vaginally delivered infants who were exclusively (crude beta-coefficient, cβ: −1.01; adjusted beta-coefficient, aβ: −1.03, *p* < 0.05, Figure 2 and Table 2) and partially breastfed (cβ: −5.73, aβ: −6.24, *p* < 0.05, Figure 2 and Table 2). CS delivery, regardless of labour, and emergency CS were also associated with reduced absolute quantities of bifidobacteria, independent of BMI (*p* < 0.05 for all infants, Table 2). BMI-adjusted associations were seen for CS with or without labour in non-breastfed infants (*p* < 0.05, Table 2). BMI adjustment revealed statistically significant reductions in absolute quantities of bifidobacteria for CS with labour or emergency CS among exclusively breastfed or non-breastfed infants (*p* < 0.05, Table 2).

**Figure 3:**
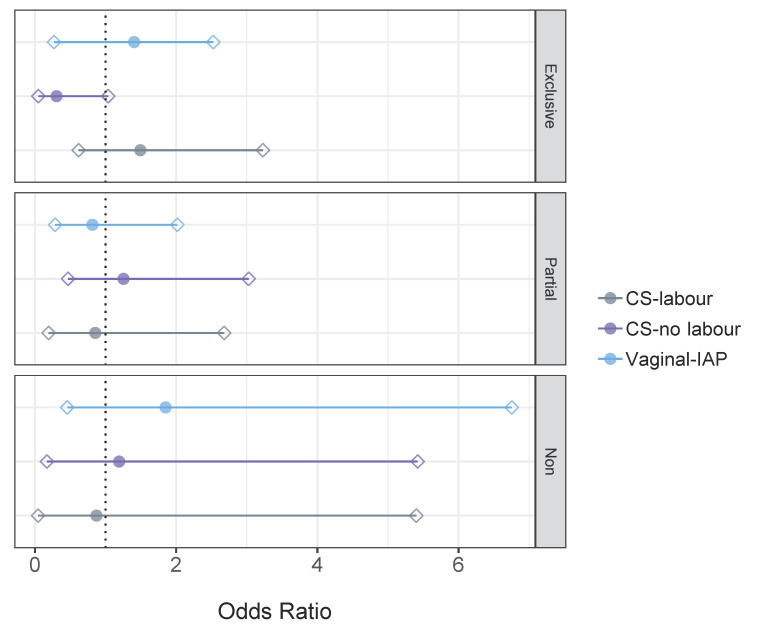
Likelihood (OR, 95% CI) of gut microbial colonization with qPCR *Bifidobacterium longum* subsp. *infantis* according to birth mode within exclusively breastfed, partially breastfed and non-breastfed infants at 3 months of age. Crude ORs from logistic regression are presented. Vaginal-no IAP is the reference group. The circle indicates the odds ratio and rhombuses indicate 95% CIs. The dotted line shows an odds ratio of 1.

*B. longum* subsp. *infantis* was detected in only 7.30% of infants. Crude odds ratios indicated no significant association between birth mode and the colonization of *B. longum* subsp. *infantis*, when stratified by feeding status (Figure 3). In addition, no associations were observed following adjustment for maternal BMI. Almost attaining statistical significance was the crude OR of 0.31 (95% CI: 0.05–1.04) for *B. longum* subsp. *infantis* following CS without labour in exclusively breastfed infants. BMI adjustment nullified this association as: OR: 0.35; 95% CI: 0.06–1.18.

### 3.2. Alpha-Diversity, Beta-Diversity of Gut Microbial Community According to a Birth Mode

Principal Coordinates Analysis (PCoA) displayed the impact of maternal IAP on the structure of infant gut microbial communities (*p*  <  0.05 according to PERMANOVA and PERMDISP; Figure S4B). In addition, alpha-diversity analyses indicated that maternal IAP exposure significantly decreased the microbial richness (observed OTUs and Chao1) (*p* < 0.001) (Figure S4A). Vaginally delivered infants exposed to maternal IAP had reduced microbial diversity (Shannon and PD whole tree) compared to unexposed infants (*p* ≤ 0.05) (Figure S4A).

### 3.3. Overall Gut Microbiota According to a Birth Mode, Stratified by Breastfeeding Status

At the phylum level, Bacteroidetes was under-represented in all IAP-exposed infants, with the most severe depletion observed in CS-delivered infants. Specifically, the average relative abundance of Bacteroidetes was 38.37% among unexposed infants, whereas it was 30.81% among vaginally born IAP-exposed infants, and less than 13.0% in CS-delivered infants (Figure S3A). In all infant feeding modes, CS with or without labour was associated with a lower abundance of Bacteroidetes and members of the genus *Bacteroides* than vaginal delivery with no IAP (Figure 1B,C,E,F,H,I). However, reduced abundance of Bacteroidetes and *Bacteroides* was solely observed following vaginal delivery with IAP in exclusively and partially breastfed infants (Figure 1A).

Maternal IAP exposure, particularly CS delivery, was associated with highly abundant Firmicutes and Proteobacteria, regardless of breastfeeding status (Figure 1 and Figure S3). Members of genera belonging to the Firmicutes, including *Enterococcus*, *“Clostridium”*, unclassified Clostridiaceae, *Veillonella*, were highly abundant in infants born by CS (Figure S5B,C). Members of the phylum Proteobacteria, such as unclassified Enterobacteriaceae, other Enterobacteriaceae, and *Citrobacter*, were most commonly found among CS-delivered infants whether they experienced labour or not (Figure S5B,C). With any breastfeeding, genus *Citrobacter* were more abundant in infant gut microbiota following IAP exposure in vaginal or CS birth (Figure 1A–F); if breastfeeding was exclusive, genus *Enterococcus* also became more abundant when compared to vaginal birth with no IAP (Figure 1A–C).

### 3.4. Co-Occurrence Network between Bifidobacterium and Other Gut Microbiota

**Figure 4:**
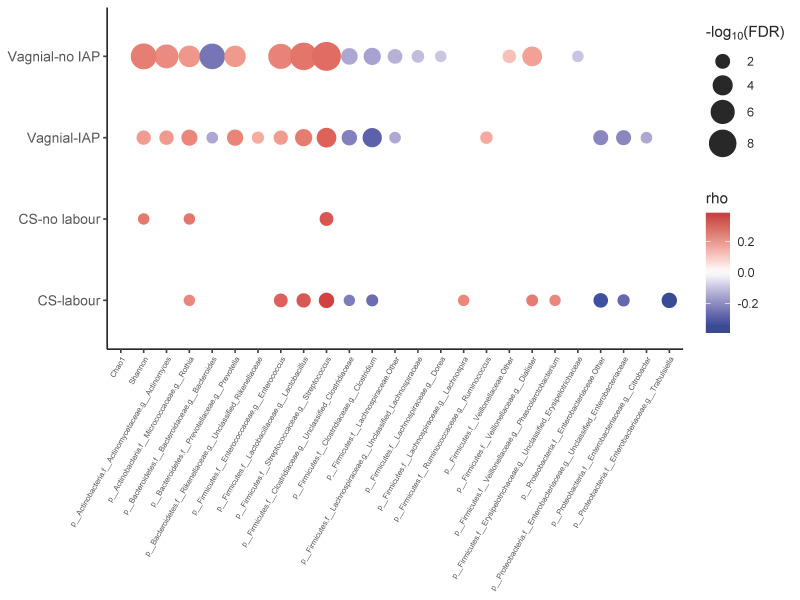
Correlation networks showing associations between *Bifidobacterium* and other gut microbial genera according to the birth mode in exclusively breastfed infants. Circle sizes and colour intensity (Red = positive, blue = negative) are proportional to the significance and strength of association, respectively. Only significant (FDR < 0.1) correlations were displayed.

Significant correlations (FDR < 0.1) between *Bifidobacterium* and other microbial taxa at the genus level among exclusively breastfed infants were identified and shown in Figure 4 (partially breastfed, Figure S6; non-breastfed, Figure S7). The genus *Bifidobacterium* had increased Shannon diversity and exhibited a strongly positive correlation with the genera *Rothia*, *Enterococcus*, *Lactobacillus* and *Streptococcus*, and a negative correlation with *“Clostridium”* and unclassified Clostridiaceae among exclusively breastfed infants following most all types of delivery mode, except CS delivery without labour (Figure 4). *Bacteroides* showed a strongly negative correlation with *Bifidobacterium* among vaginally delivered infants, especially those without IAP exposure (Figure 4). Members of the Proteobacteria, including species of unclassified and other Enterobacteriaceae, were negatively correlated with *Bifidobacterium* in infants delivered vaginally with IAP and by CS with labour (Figure 4).

## 4. Discussion

In a general population birth cohort of 1654 infants, maternal IAP exposure was associated with reduced absolute quantities of bifidobacteria among vaginally delivered infants (6.80 vs. 7.14 log_10_ (gene-copies/g faeces), *p* < 0.05), as well as their lowered abundance relative to other gut microbiota. These results are consistent with a systematic review of eight studies showing depletion of bifidobacteria in the gut microbiota of infants exposed to intrapartum antibiotics, although this difference did not persist to one month after birth in three studies [41]. They were also independent of maternal pre-pregnancy BMI, a determinant of bifidobacterial abundance in breast milk [42]. We also found depletion of bifidobacteria in caesarean birth, with or without labour, compared to vaginal delivery without IAP. This finding too, was observed in several studies until an infant age of 6 months [4], and more obviously in the early weeks after birth [3]. In CHILD Cohort Study women, GBS and caesarean prophylaxis were primarily implemented through intrapartum administration of penicillin and cefazolin, respectively [43]. All species and strains of *Bifidobacterium* are sensitive to penicillin antibiotics but less so to the cephalosporins [44]. In fact, each additional hour of IAP exposure, namely to penicllin, is reportedly associated with a 7% reduction in the abundance of bifidobacteria in infant gut microbiota [13]. Since breast milk is a rich source of HMOs that act as “prebiotics” to promote the growth of bifidobacteria [45,46], the extent of breastfeeding by delivery type is a candidate explanation for IAP differences. Certainly, the gut microbiota of exclusively breastfed vaginally delivered infants contains the highest amounts of bifidobacteria soon after birth [47]. However, when comparisons were conducted within infant feeding strata in our study, statistically significant reductions in bifidobacterial quantity were solely observed between IAP and no IAP in vaginally delivered infants who were exclusively (6.83 vs. 7.14 log_10_ (gene-copies/g faeces), *p* < 0.05) or partially breastfed (6.75 vs. 7.25 log_10_ (gene-copies/g faeces), *p* < 0.05), even when adjusted for pre-pregnancy BMI. In the next paragraphs, we turn to findings on correlations between *Bifidobacterium* and other microbiota, as proxy measures for cooperation and competition among gut microbiota, to speculate on the meaning of the study’s findings.

While of lesser importance than IAP in modifying whole gut microbial composition in the initial months after birth, breastfeeding plays a greater role in shaping gut microbiota with advancing age [7]. The co-existence *Bifidobacterium* species with other intestinal microbes was attributed to survival strategies adopted by bifidobacteria, including glycan-harvesting, glycan-breakdown and cross-feeding behaviour [48]. Specifically, bifidobacteria are able to digest breast milk HMOs into small sugars, which are further metabolized by glycolytic microbes such as species of *Streptococcus* and *Enterococcus* [49]. This metabolic cross-feeding likely accounts for the positive correlation between *Bifidobacterium* and *Streptococcus* or *Enterococcus* in our study, as does their co-occurrence in 3-month human milk of CHILD study infants [50]. Similar to results reported by others [51], we observed an inverse correlation between *Bifidobacterium* and *Bacteroides* at 3 months of age, but solely in vaginally delivered infants. This was especially apparent in the absence of IAP, when *Bifidobacterium* and *Bacteroides* were both highly abundant and competing for HMOs [52]. Members of the phylum Proteobacteria were also inversely correlated with *Bifidobacterium*, the former of which were most enriched in exclusively breastfed infants delivered by CS with labour. Because bifidobacteria produce organic acids and generate a low pH environment [53], as well as producing antimicrobial polysaccharides [54], they create unfavorable environments for other gut microbiota. Altogether, vaginal IAP and caesarean section promote Proteobacterial blooms, propagating antibiotic resistance genes and giving these pathobionts a competitive advantage; in the case of IAP, the growth of *Bifidobacterium* is also inhibited [55]. Since gut microbiota of infants born following vaginal IAP are not depleted with Bacteroidetes species to nearly the same extent as after caesarean birth, their continued presence may further reduce bifidobacterial quantities as they compete for HMOs.

Three months after birth, we observed gut microbial communities following vaginal IAP or caesarean birth to be distinct from undisturbed vaginal birth in terms of beta-diversity and lowered OTUs and species richness. Common across IAP and both CS types was over-representation by genera in the Clostridiaceae and Enterobacteriaceae families. Genus *Veillonella* was more abundant among CS-delivered infants. All of these findings were shown to be characteristic of CS birth in a recent meta-analysis published by Podlesny et al. [56]. Non-breastfed infants had lower absolute quantities of bifidobacteria at 3 months, especially if they were delivered by CS with or without labour. CS with labour differences were also statistically significant in exclusively breastfed infants; this is putatively a function of reduced bifidobacterial abundance with prolonged labour [10]. Mitchell, et al. [1] and Shao, et al. [7] noted a similar gut microbial composition in 2–3 week old infants born by post-labour or pre-labour CS, including one with depleted *Bacteroides*, but no appreciable changes to the relative abundance of *Bifidobacterium*. In our study, neither the absolute nor the relative *Bifidobacterium* quantity differed among the CS definition types: CS without labour vs. elective CS or CS with labour vs. emergency CS. However, relative abundance of *Bifidobacterium* was higher following elective CS compared to emergency CS delivery. Beta-diversity differences in infant gut microbiota between these clinically defined CS births were also found [57]. In fact, relative to vaginal birth with no IAP, we noted the reduction in bifidobacteria to be more robust for emergency CS than for CS with labour. When cases of pre-labour emergency CS were re-classified as CS without labour, *Bifidobacterium* differences were no longer evident between CS with or without labour. Prenatal complications, such as preeclampsia, a common indication for pre-labour emergency CS, may lead to reduced bifidobacteria in maternal gut microbiota of the third trimester [58] and to the transfer of bifidobacteria to the newborn.

There are several strengths of this study. Firstly, the CHILD cohort is a well-characterized birth cohort, such that we were able to investigate in-depth the impact of IAP and birth mode on infant gut microbiota. Secondly, an adequate sample size allowed us to adjust for important confounding factors, particularly breastfeeding. Last but not least, the qPCR-based abundance data were complementary to the 16S-based relative abundance data typically analysed in many microbiome studies. Although our Illumina MiSeq method was cost-effective for the sequencing of the 16S rRNA gene in a large number of faecal samples, several studies noted the under-representation of the genus *Bifidobacterium* when a universal primer set targeting the V4 region was employed. This study also used genus- and species-specific primer sets to quantify faecal *Bifidobacterium* and *B. longum* subsp. *infantis* to improve detection of the bifidobacteria. One limitation of this study was that we assayed only one species of *Bifidobacterium* (i.e., *B. longum* subsp. *infantis*) using the species-specific qPCR.

## 5. Conclusions

In conclusion, this study revealed the impact of the birth mode on the bifidobacterial abundance in infant gut microbiota after 3 months. Specifically, we found that maternal IAP exposure during vaginal birth significantly decreased the absolute and relative abundance of *Bifidobacterium,* even among breastfed infants. Moreover, absolute quantities of bifidobacteria were negatively affected by CS birth, with or without labour. Our epidemiological study points to the important ecological role of the genus *Bifidobacterium* in terms of its interaction with other gut microbiota during early infancy.

## Figures and Tables

**Table 1:** Primers used in this study.

Target Microorganism	Primer Set/Probe Name	Sequence (5′–3′)	References
Total bacteria	Forward (Total F)	TCCTACGGGAGGCAGCAGT	[34]
	Reverse (Total R)	GGACTACCAGGGTATCTAATCCTGTT	
	Probe (Total Probe)	5′6-FAM /CGTATTACCGCGGCTGCTGGCAC/3′TAMRA	
*genus Bifidobacterium*	Forward (Bif F)	GCGTGCTTAACACATGCAAGTC	[33]
	Reverse (Bif R)	CACCCGTTTCCAGGAGCTATT	
	Probe (Bif probe)	5′TexRd-XN/TCACGCATTACTCACCCGTTCGCC/3′IAbRQSp	
*B. longum* subsp. *infantis*	Forward (Blon0915-F)	CGTATTGGCTTTGTACGCATTT	[33]
	Reverse (Blon0915R)	ATCGTGCCGGTGAGATTTAC	
	Probe (Blon0915 Probe)	5′6-FAM/CCAGTATGG/ZEN/CTGGTAAAGTTCACTGCA/3′IABkFQ	

**Table 2:** Associations between birth mode and the absolute quantity of genus *Bifidobacterium* by qPCR analysis, all and stratified by feeding status ^†^.

**CS Groups Defined in This Study**							
**Breastfeeding Status**	**Linear Regression Model**	**Vaginal-IAP**		**CS-No Labour**		**CS-Labour**	
		**Beta-Coefficient** **(95% CI)**	***p* Value**	**Beta-Coefficient** **(95% CI)**	***p* Value**	**Beta-Coefficient** **(95% CI)**	***p* Value**
All	Crude	−1.12 (−1.75, −0.49)	**0.0005**	−0.98 (−1.78, −0.19)	**0.02**	−0.88 (−1.75, −0.01)	**0.047**
	Adjusted ^‡^	−1.18 (−1.83, −0.52)	**0.0004**	−1.05 (−1.87, −0.22)	**0.01**	−1.29 (−2.19, −0.39)	**0.005**
Exclusive	Crude	−1.01 (−1.88, −0.13)	**0.02**	−0.66 (−1.89, 0.57)	0.29	−1.03 (−2.27, 0.21)	0.10
	Adjusted	−1.03 (−1.93, −0.13)	**0.03**	−0.91 (−2.19, 0.38)	0.17	−1.31 (−2.58, −0.04)	**0.04**
Partial	Crude	−5.73 (−10.00, −1.46)	**0.01**	−3.96 (−8.79, 0.87)	0.11	−2.09 (−7.74, 3.55)	0.47
	Adjusted	−6.24 (−10.72, −1.77)	**0.01**	−3.94 (−8.91, 1.03)	0.12	−2.86 (−8.66, 2.94)	0.33
Non	Crude	−0.86 (−2.26, 0.55)	0.23	−1.77 (−3.40,−0.15)	**0.03**	−1.02 (−2.95, 0.91)	0.30
	Adjusted	−0.91 (−2.32, 0.50)	0.20	−1.76 (−3.43, −0.09)	**0.04**	−2.59 (−4.61, −0.56)	**0.01**
**CS Groups Defined Clinically**							
**Breastfeeding Status**	**Linear Regression Model**	**Vaginal-IAP**		**CS-Elective**		**CS-Emergency**	
		**Beta-Coefficient** **(95% CI)**	***p* Value**	**Beta-Coefficient** **(95% CI)**	***p* Value**	**Beta-Coefficient** **(95% CI)**	***p* Value**
All	Crude	−1.12 (−1.75, −0.49)	**0.0005**	−0.79 (−1.71, 0.13)	0.09	−1.04 (−1.80, −0.27)	**0.01**
	Adjusted	−1.18 (−1.83, −0.52)	**0.0004**	−0.88 (−1.84, 0.08)	0.07	−1.33 (−2.13, −0.54)	**0.001**
Exclusive	Crude	−1.01 (−1.88, −0.13)	**0.02**	0.18 (−1.30, 1.65)	0.82	−1.34 (−2.43, −0.25)	**0.02**
	Adjusted	−1.03 (−1.93, −0.13)	**0.03**	−0.22 (−1.78, 1.33)	0.78	−1.52 (−2.65, −0.40)	**0.01**
Partial	Crude	−5.73 (−9.99, −1.47)	**0.01**	−5.74 (−11.01, −0.47)	**0.03**	−0.92 (−5.98, 4.14)	0.72
	Adjusted	−6.24 (−10.71, −1.77)	**0.01**	−5.72 (−11.17, −0.27)	**0.04**	−1.54 (−6.73, 3.66)	0.56
Non	Crude	−0.86 (−2.26, 0.55)	0.23	−1.45 (−3.31, 0.42)	0.13	−1.50 (−3.16, 0.17)	0.08
	Adjusted	−0.91 (−2.32, 0.49)	0.20	−1.25 (−3.17, 0.68)	0.20	−2.71 (−4.42, −0.99)	**0.002**

^†^ Analysis is conducted by univariable and multivariable linear regression. To improve the normality, the absolute quantity of genus *Bifidobacterium* is Box–Cox transformed. Vaginal-no IAP is the reference group. Significant *p* values are shown in bold. ^‡^ Model is adjusted for maternal BMI.

## Data Availability

Restrictions apply to the availability of these data. Data was obtained from the CHILD Cohort Study and are available via childcohort.ca (accessed on 1 May 2021) with the permission of Anita Kozyrskyj and the Child Cohort Study National Coordinating Centre.

## Supplementary Materials

The following are available online at https://www.mdpi.com/article/10.3390/microorganisms9091847/s1, Figure S1: Directed acyclic graph (DAG) of the association between the birth mode and infant gut microbiota at three months of age, Figure S2: Scatter plots with the linear regression line demonstrating the relationship between qPCR- and 16S-based relative abundance (A); and the relationship between qPCR-based absolute abundance and 16S-based relative abundance (B) for *Bifidobacterium* in infants aged three months, Figure S3: Relative abundance of dominant phyla (A) and genera (B) in infant gut microbiota at 3 months, according to the birth mode, Figure S4: Alpha-diversity (A) and beta-diversity (B) of gut microbial community in infants at 3 months in relation to the birth mode, Figure S5: Linear discriminant analysis of gut microbiota according to birth mode, comparing vaginal-IAP (A), CS-no labour (B), CS-labour (C), CS-elective (D), and CS-emergency (E) vs. vaginal-no IAP at 3 months, Figure S6: Correlation networks showing associations between *Bifidobacterium* and other gut microbial genera according to the birth mode in partially breastfed infants, Figure S7: Correlation networks showing associations between *Bifidobacterium* and other gut microbial genera according to the birth mode in non-breastfed infants, Table S1: Maternal and infant characteristics stratified by birth mode, Table S2: Median relative abundance of genus *Bifidobacterium* in faecal microbiota of infants according to a birth mode at 3 months by 16S rRNA sequencing and qPCR analysis.
