# Supplementary material for: Impact of Maternal Intrapartum Antibiotics, and Caesarean Section with and without Labour on Bifidobacterium and Other Infant Gut Microbiota
Source: Microorganisms. 2021 Aug 31;9(9):1847. doi: 10.3390/microorganisms9091847 (PMC8467529; doi:10.3390/microorganisms9091847)
Supplement: Supplementary file 1 [file microorganisms-09-01847-s001.zip › Figure S1-S7.pptx]

## Slide 1
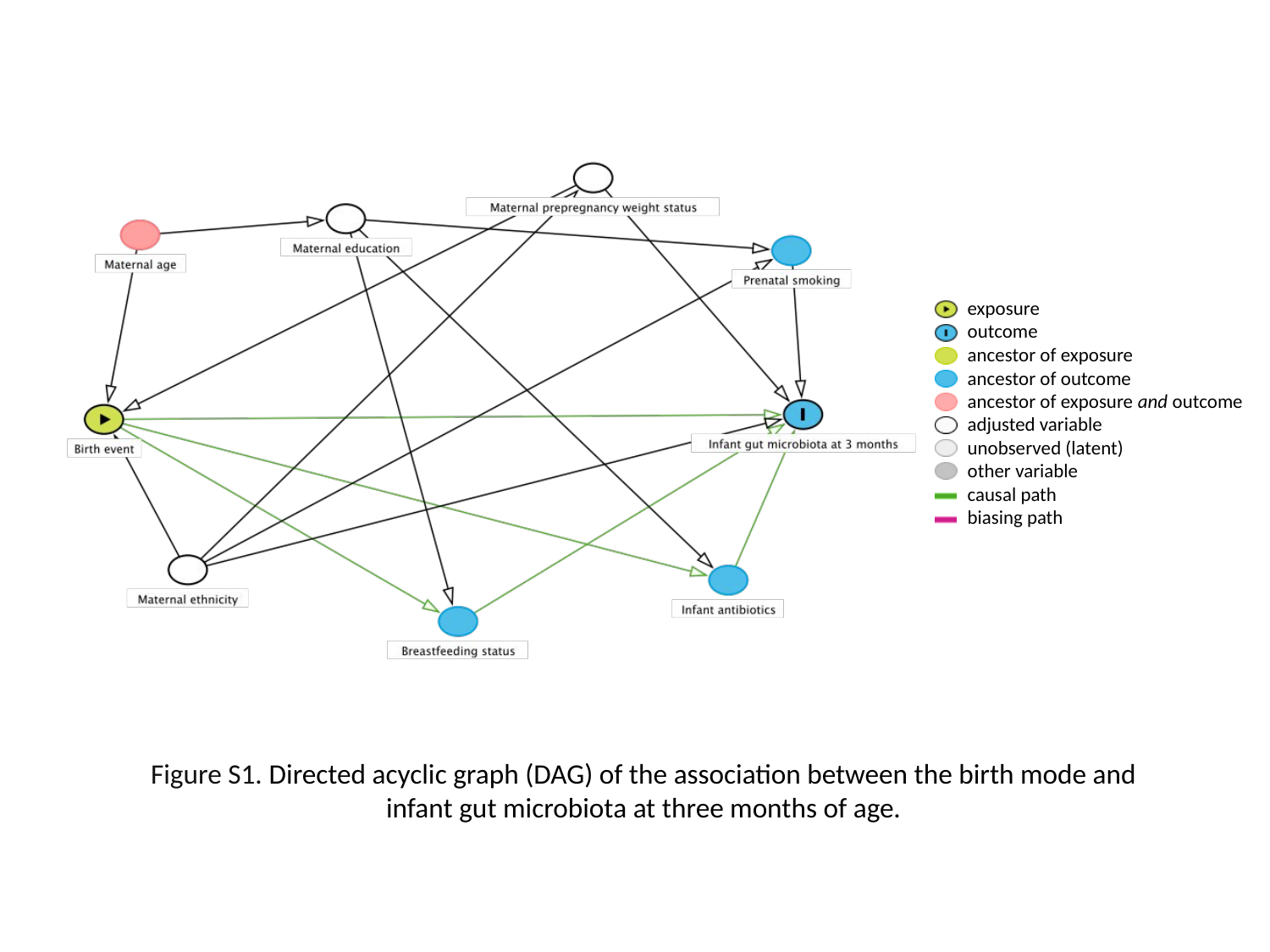

exposure
  outcome
  ancestor of exposure
  ancestor of outcome
  ancestor of exposure and outcome
  adjusted variable
  unobserved (latent)
  other variable
  causal path
  biasing path
Figure S1. Directed acyclic graph (DAG) of the association between the birth mode and infant gut microbiota at three months of age.

## Slide 2
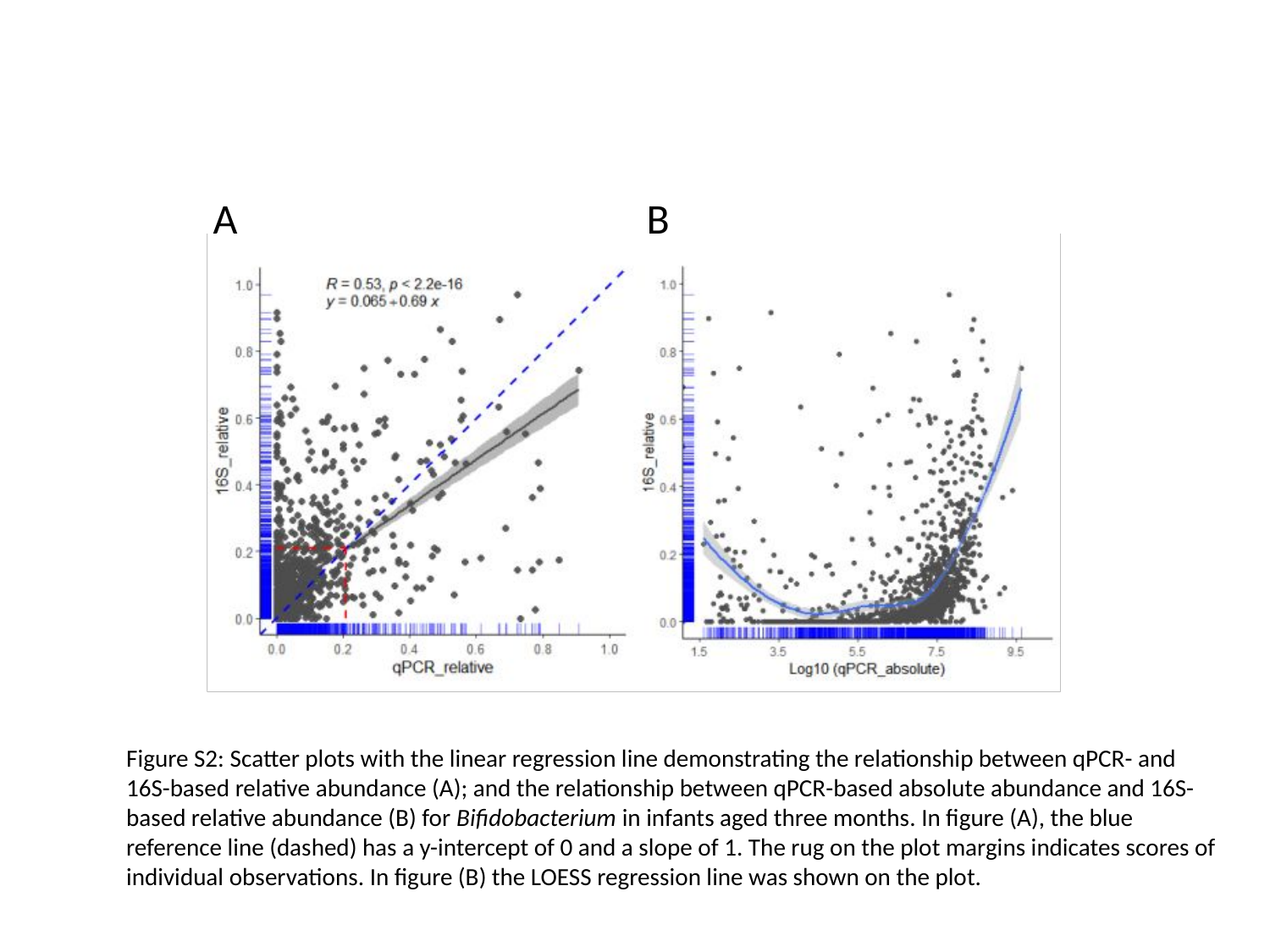

B
A
Figure S2: Scatter plots with the linear regression line demonstrating the relationship between qPCR- and 16S-based relative abundance (A); and the relationship between qPCR-based absolute abundance and 16S-based relative abundance (B) for Bifidobacterium in infants aged three months. In figure (A), the blue reference line (dashed) has a y-intercept of 0 and a slope of 1. The rug on the plot margins indicates scores of individual observations. In figure (B) the LOESS regression line was shown on the plot.

## Slide 3
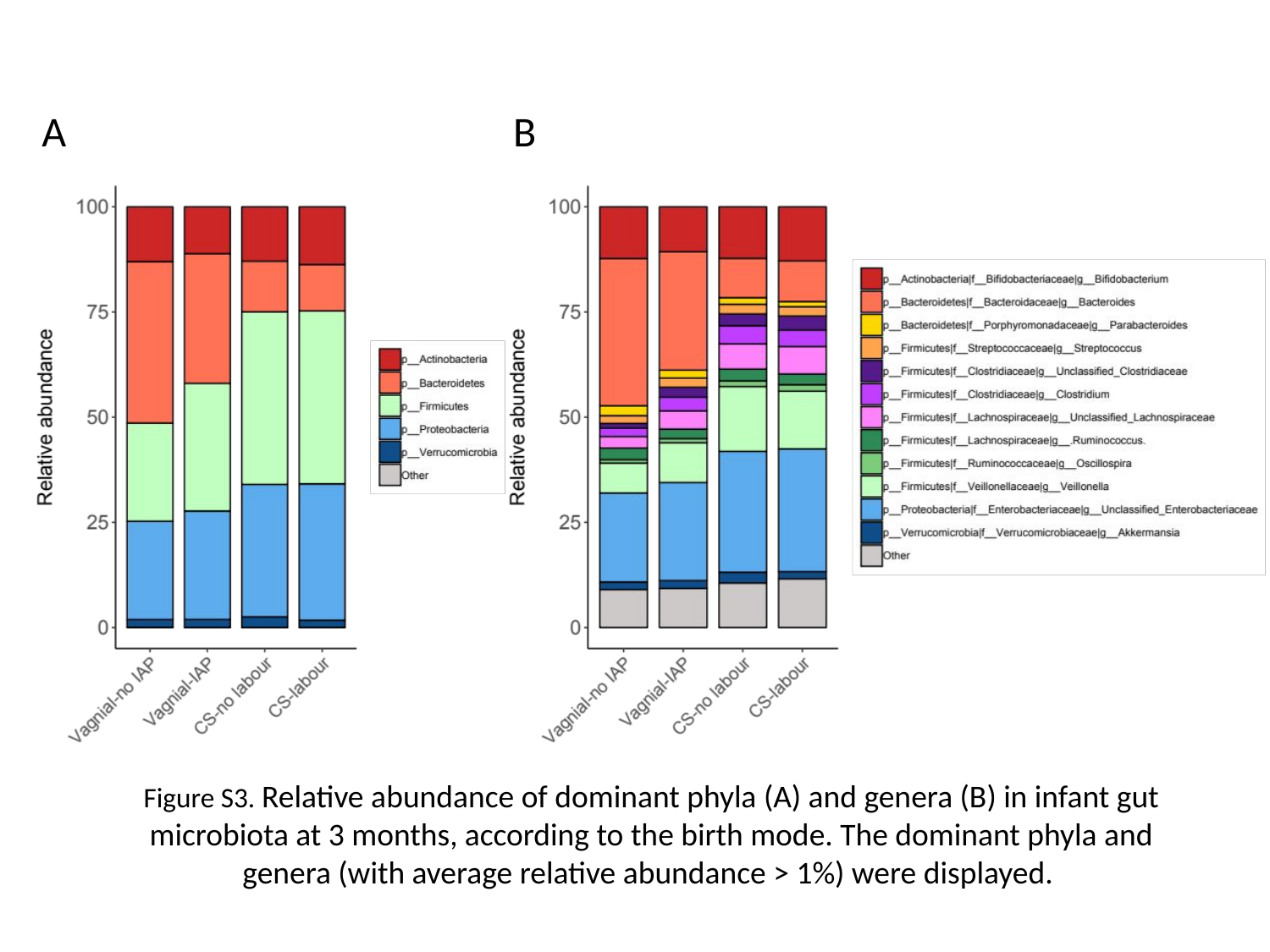

A
B
Figure S3. Relative abundance of dominant phyla (A) and genera (B) in infant gut microbiota at 3 months, according to the birth mode. The dominant phyla and genera (with average relative abundance > 1%) were displayed.

## Slide 4
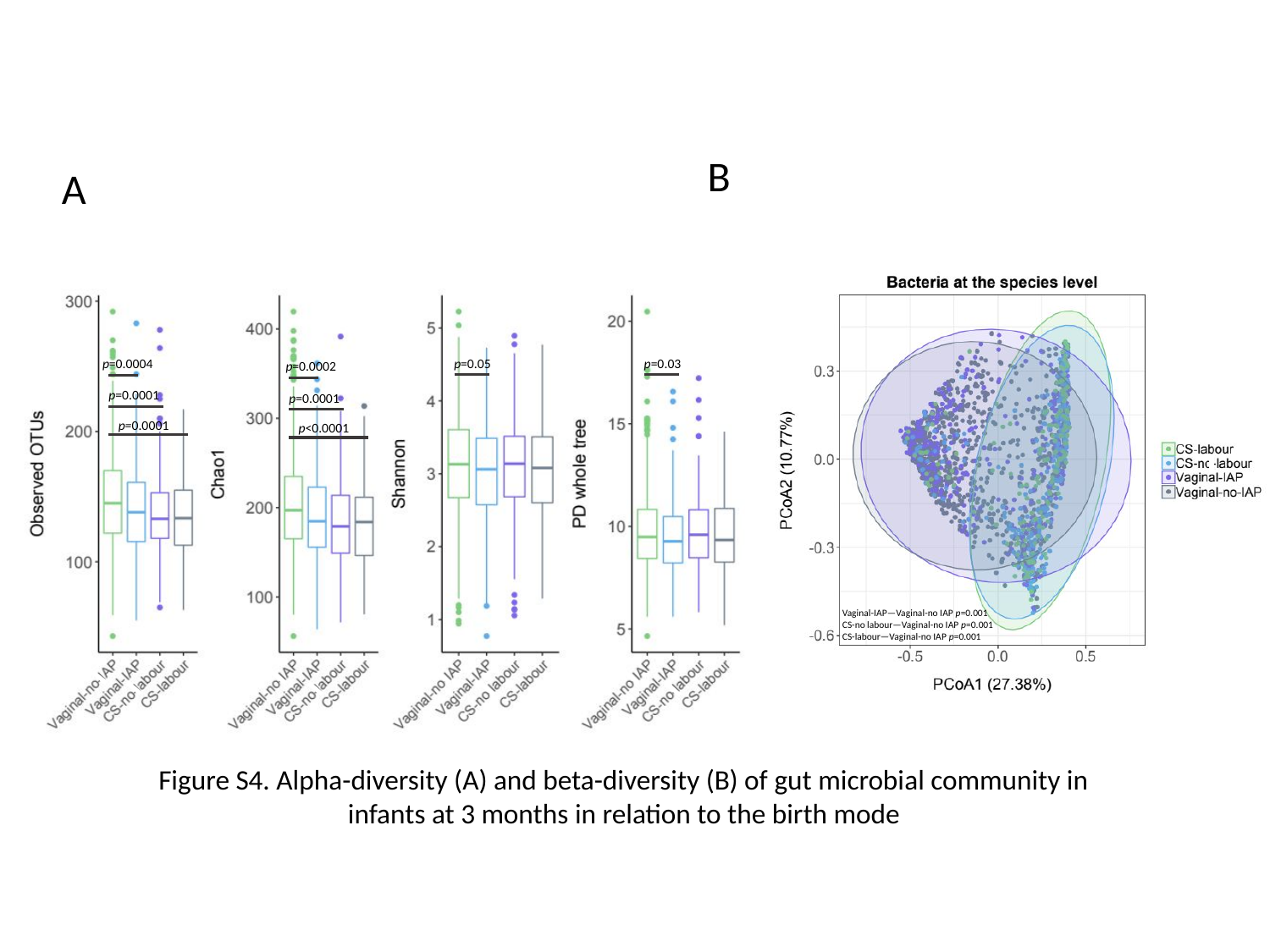

B
A
Vaginal-IAP—Vaginal-no IAP p=0.001
CS-no labour—Vaginal-no IAP p=0.001
CS-labour—Vaginal-no IAP p=0.001
p=0.0004
p=0.05
p=0.03
p=0.0002
p=0.0001
p=0.0001
p=0.0001
p<0.0001
Figure S4. Alpha-diversity (A) and beta-diversity (B) of gut microbial community in infants at 3 months in relation to the birth mode

## Slide 5
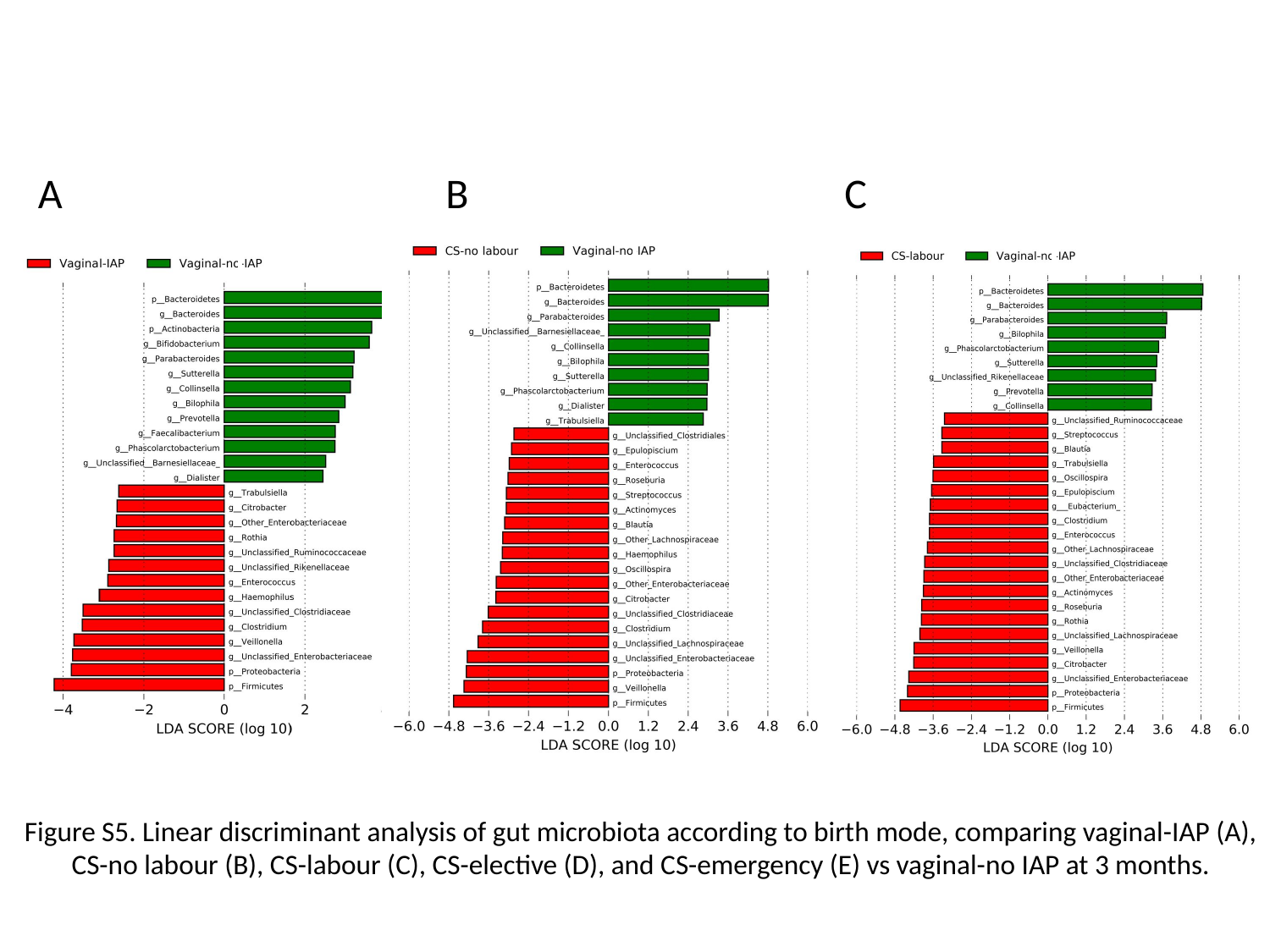

A
B
C
Figure S5. Linear discriminant analysis of gut microbiota according to birth mode, comparing vaginal-IAP (A), CS-no labour (B), CS-labour (C), CS-elective (D), and CS-emergency (E) vs vaginal-no IAP at 3 months.

## Slide 6
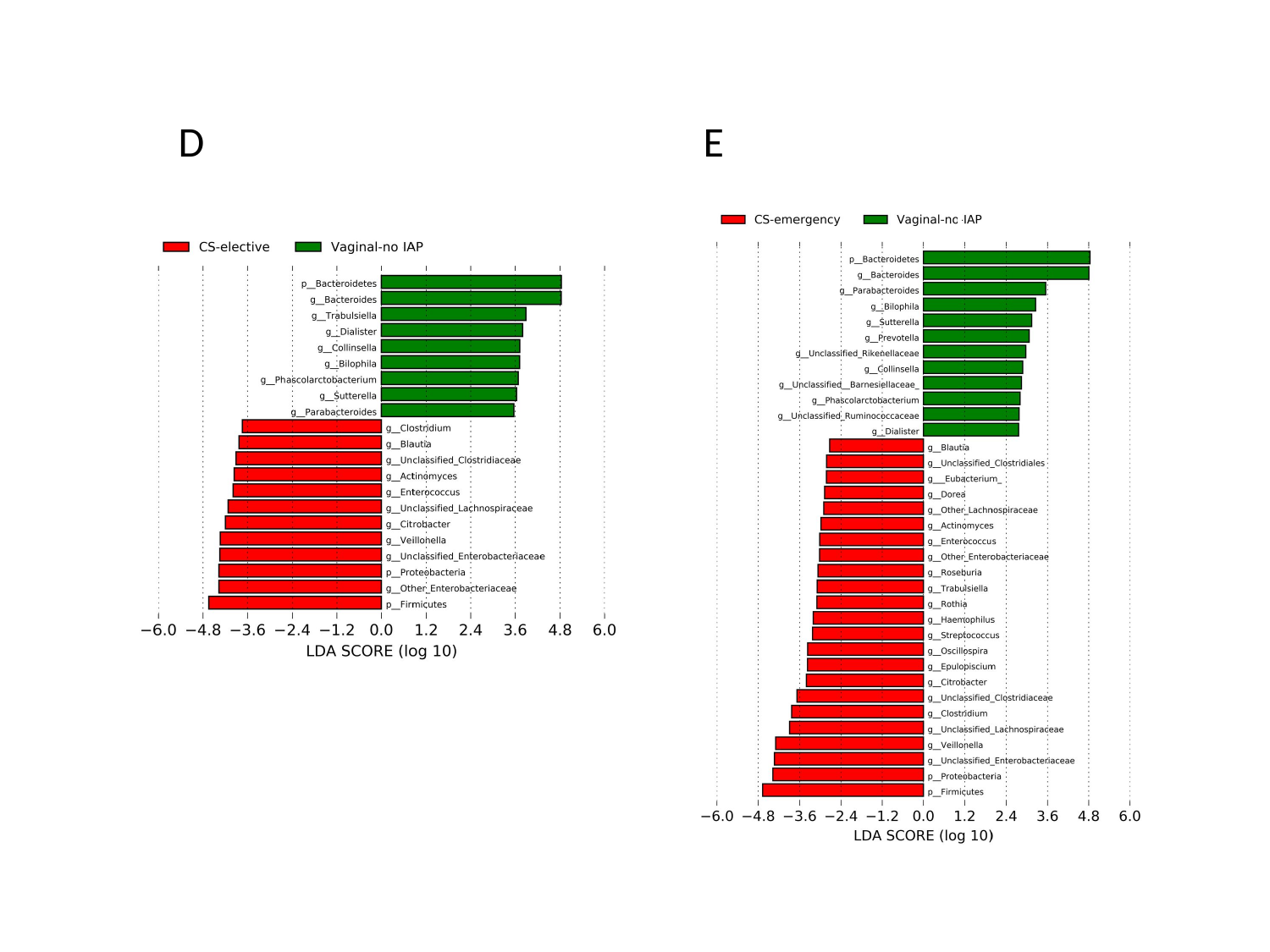

D
E

## Slide 7
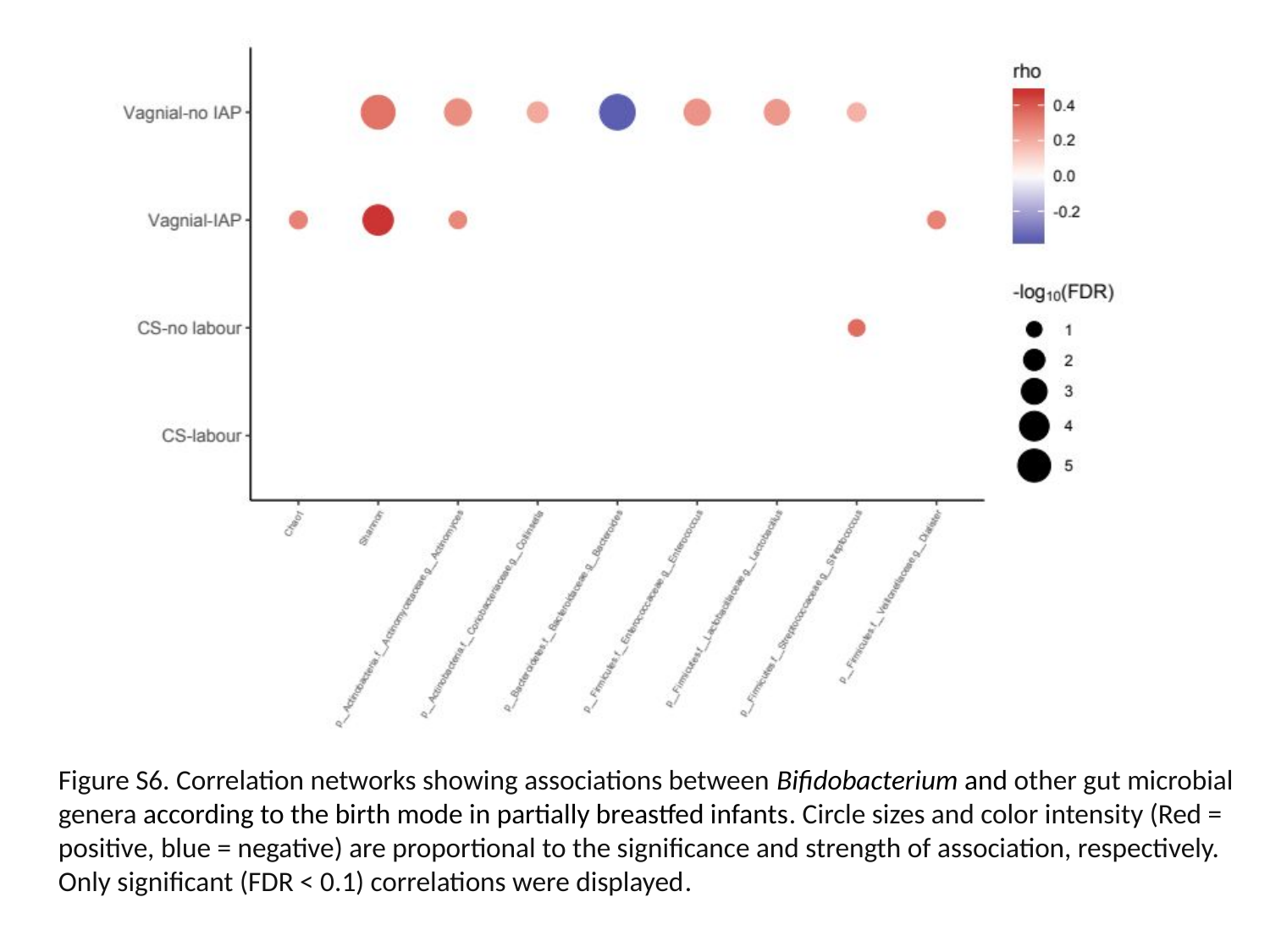

Figure S6. Correlation networks showing associations between Bifidobacterium and other gut microbial genera according to the birth mode in partially breastfed infants. Circle sizes and color intensity (Red = positive, blue = negative) are proportional to the significance and strength of association, respectively. Only significant (FDR < 0.1) correlations were displayed.

## Slide 8
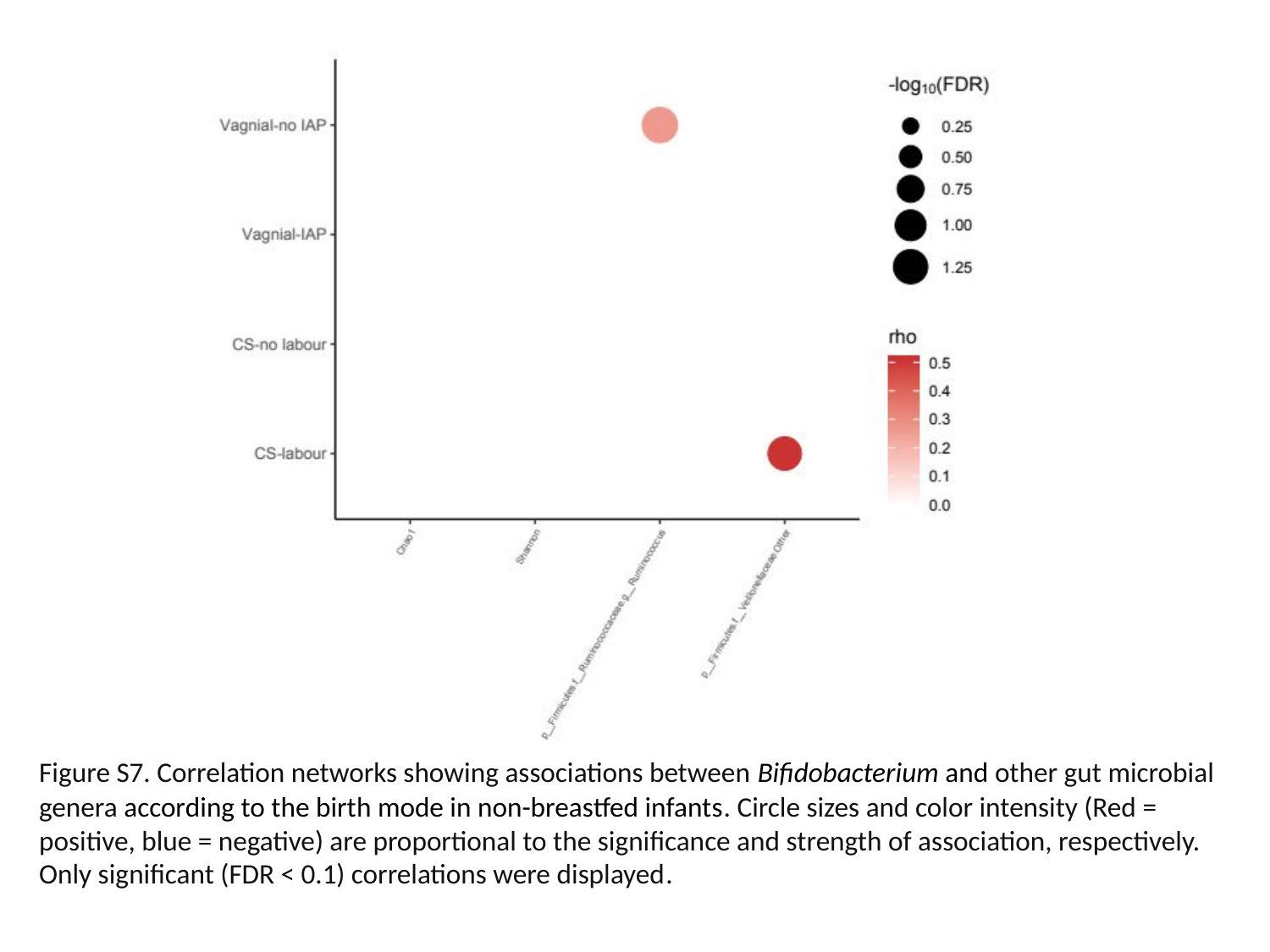

Figure S7. Correlation networks showing associations between Bifidobacterium and other gut microbial genera according to the birth mode in non-breastfed infants. Circle sizes and color intensity (Red = positive, blue = negative) are proportional to the significance and strength of association, respectively. Only significant (FDR < 0.1) correlations were displayed.
